# Supplementary figures and images for: PGC-1α activation to enhance macrophage immune function in mycobacterial infections
Source: PLoS One. 2025 Feb 6;20(2):e0310908. doi: 10.1371/journal.pone.0310908 (PMC11801632; doi:10.1371/journal.pone.0310908)

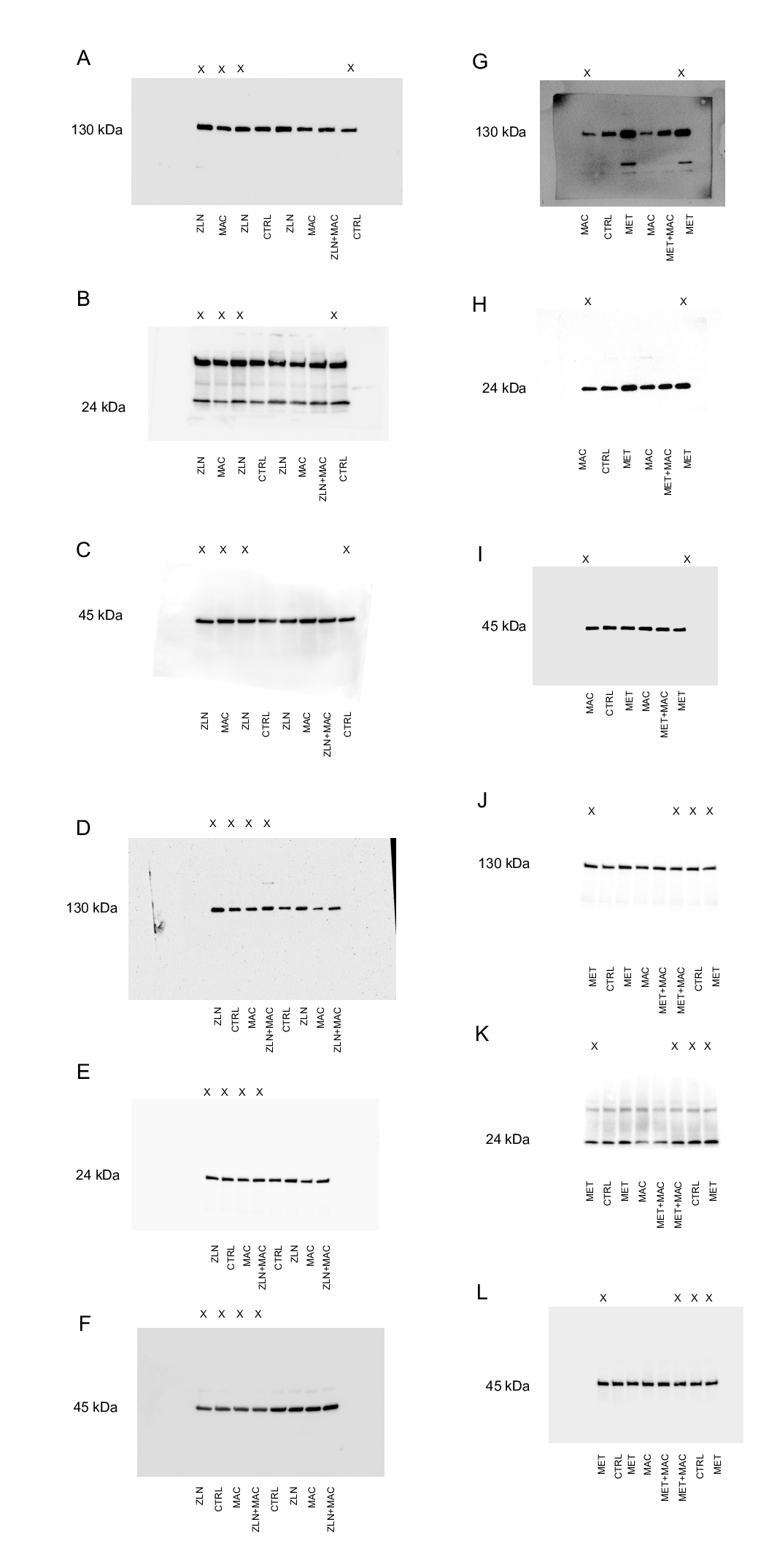

Supplement: S1 Raw images — Raw Western blot images from the manuscript. Blots were incubated with primary antibodies and HRP-conjugated secondary antibodies and acquired through chemiluminescence imaging. Fig 2: PGC-1α (A, D), TFAM (B, E), β-Actin (C, F); Fig 3: PGC-1α (G, J), TFAM (H, K), β-Actin (I, L). (TIF) [file pone.0310908.s001.tif]

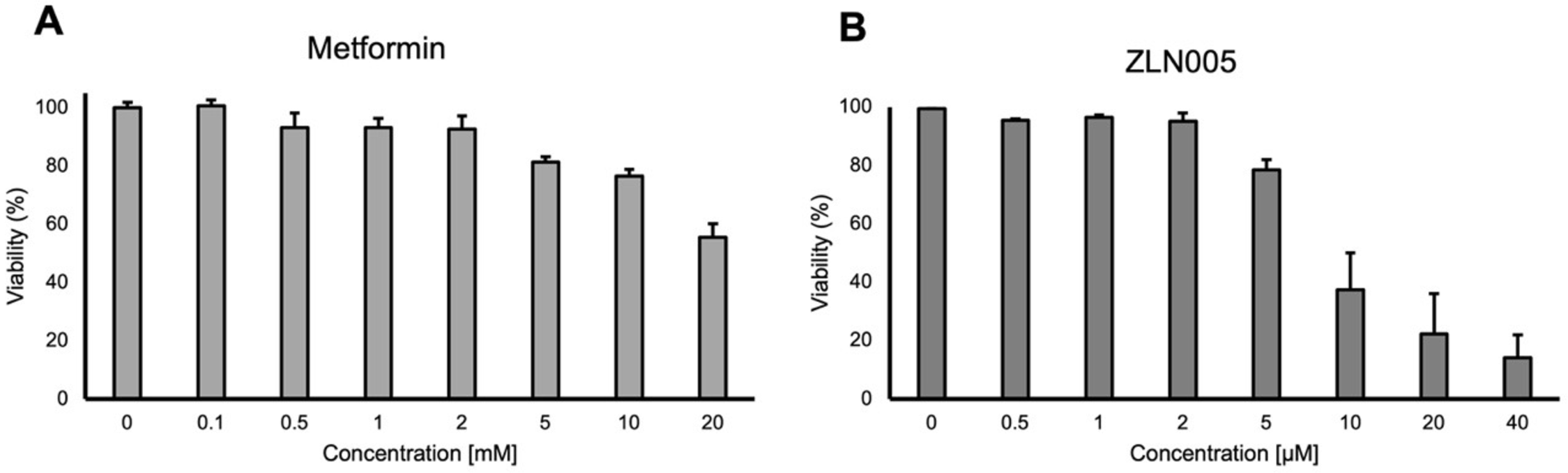

Supplement: S1 Fig — THP-1 macrophages were treated with a range of concentrations of metformin (A) and ZLN005 (B) for 24 hours. An MTT assay was utilized to determine toxicity and the optimal drug concentration for treatment of cells. (TIF) [file pone.0310908.s002.tif]

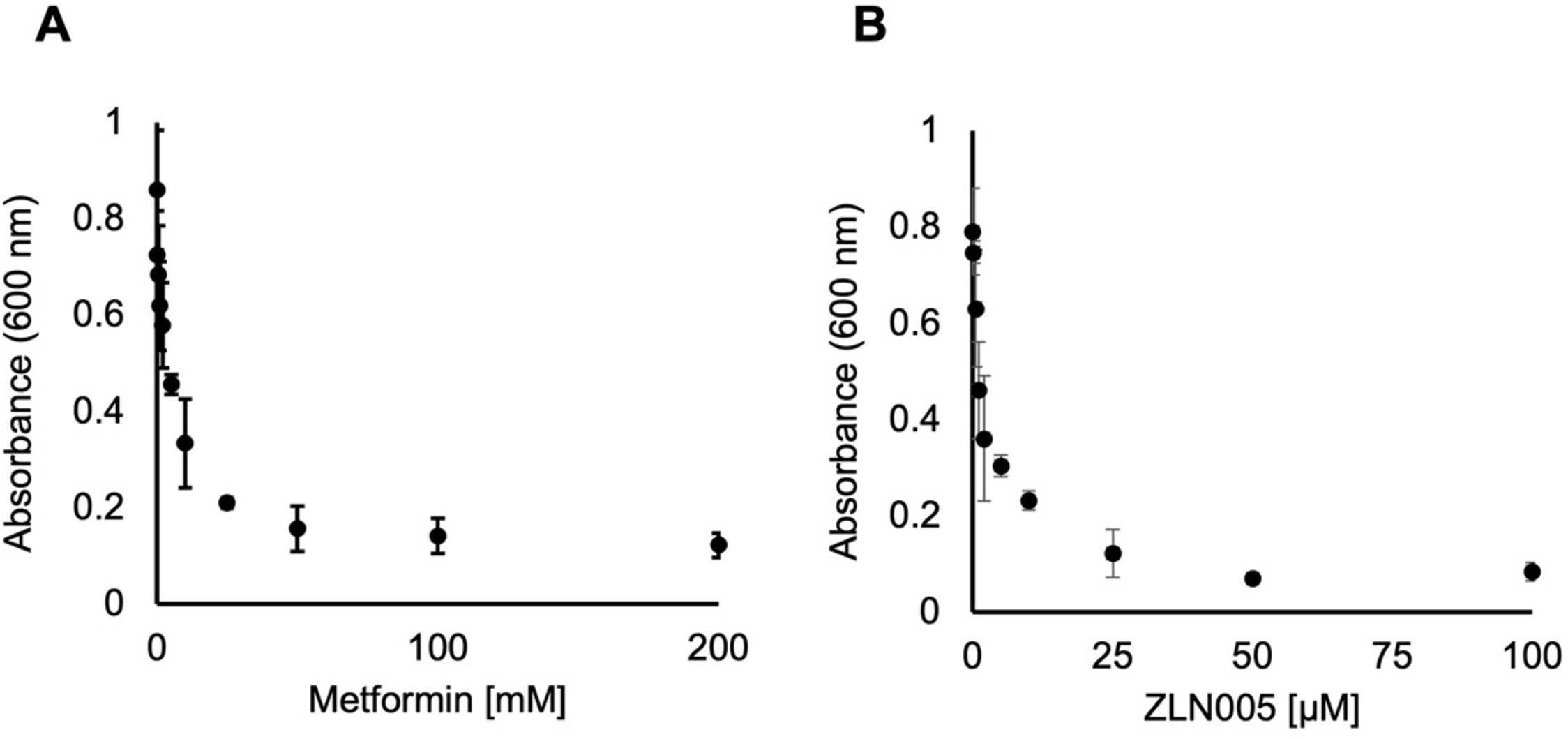

Supplement: S2 Fig — MAC cultures were treated with a range of concentrations of metformin (A) and ZLN005 (B) for 24 hours, and the OD600 was measured to determine toxicity to MAC. Concentrations used in cell culture treatments was found to have no significant influence on MAC in vitro. (TIF) [file pone.0310908.s003.tif]
