# Supplementary material for: PGC-1α activation to enhance macrophage immune function in mycobacterial infections
Source: PLoS One. 2025 Feb 6;20(2):e0310908. doi: 10.1371/journal.pone.0310908 (PMC11801632; doi:10.1371/journal.pone.0310908)
Supplement: Attachment — Submitted filename: Response to Reviewers PlosOne April 1 2024.pdf [file pone.0310908.s005.pdf]

Response to Reviewers:

Reviewer #1:

Comment: The revised manuscript has been greatly improved. However, the in vivo studies can not prove that the altered expression of PGC-1α or TFAM, caused by NTM, occurred in macrophages.

Response: We agree with the reviewer and have now removed the in vivo data and are working on more in details studies. This manuscript will focus on the in vitro mechanistic studies.

Reviewer #2: I thank the authors for their responses.

Comment: Line 152 (revised version): What dose of oral metformin in an average human would be equivalent to the final concentration of 2 mM metformin mentioned in the manuscript. Please add to the manuscript. Appreciate authors' comments. It would be a plus for the manuscript if their explanation could be incorporated into the manuscript.

Response: Appreciate the reviewer bringing up the issue of dose of Metformin. The maximum dose of oral metformin in clinical practice is 2.5–3 g per day, which is approximately 35–42 mg/kg. In humans, the plasma concentration range of metformin after passing through the liver is usually 10–40 μM. For an invitro dose, 2 mM is would be comparable to the human doses on the higher side. This explanation has been added to the discussion of the manuscript.

Comment: Line 305 (revised version): Please add what the cells are treated with.

Response: We have now added the cells. "THP-1 cells were cultured, treated with metformin [2 mM], ZLN005 [2 μM], and SR [10 μM], and infected in 96-well plates (Grenier Bio-One) as previously described."

Comment: Figure 1C and 1D: I think there should be a mistake as the picture appears to be the same and not replaced (please see below).
a. The MitoSOX staining for CTRL seems to be nothing (at least not visible on the provided image) where as mentioned as about 1 on Figure 1D. Appreciated comment clarification.
b. MitoSox-based flow cytometry could be considered as an alternative.

Response: We appreciate the comment and regret the oversight. Figure 1C was updated to include a new image panel for the Control group, and 1D was revised with updated statistical analysis. The Control group exhibited low expression of MitoSOX staining in all replicates used for analysis. This was seen in multiple experiments performed.

Comment: Figure 2: I appreciated author's comments. As mentioned in the original comments showing the changes specifically in macrophages (by cell sorting or staining) is very important given the focus of this manuscript and the author's prior work. These experiments should be very straightforward and will significantly improve the manuscript. However, if further experiments are not feasible or can't be done then this section could be omitted particularly given that the per authors the in vivo studies are beyond the scope of this manuscript or at least the limitation clearly mentioned.

Response: We appreciate the reviewer comments and have now opted to remove the in vivo data.

Comment: Lines 434-436: Language needs revision for better clarity.
Response: This was related to the in vivo data, which has been removed, the paragraph has been revised accordingly.

Comment: Figure 4 related:
a. Text mentions PGC-1a while figure A and B mention “ppargc1a” (e.g., line 440 could change to “decreases in PGC1-a mRNA (ppargc1a)”.
b. Figure 4 (K, L, M, N): While figures K and L mention “ZLN + MAC”, figures M and N and the text mention MET + MAC. Which one is it? The correct image should be MET + MAC to confirm to the text and the rest of images. Please rectify.

Response:

a. The text was edited to specify the protein and RNA forms of PGC-1α and TFAM; PGC-1α mRNA as “ppargc1a” and TFAM mRNA as “tfam”

b. The figures are edited to add the proper treatment conditions.

Comment: Figure 5: Again:
a. SR (I assume stands for SR-18292) is mentioned in the figure but no mention or explanation of SR in either the text or the figure legend.
b. Although SR is mentioned on line 510 (and not 520) and under next topic and in relation to Figure 6, it should be mentioned at the first appearance for clarity.

Response:

Appreciate the comment, regret the oversight. This has been corrected. SR-18292 is referred to as “SR” first in the materials and methods (line 154), and in the main text body (line 540)

Comment: Figure 6:
a. A and B. Moving rows 7 to just above current row 4 (MAC) will help the clarity in relative to C and D.
b. Also, the legend incorporated in the figures C and D needs to be adjusted to incorporate explanation for the “infection” part of the images.

Response: The image panels were adjusted for better clarity and organization. The figure legend was updated to include “Uninfected” and “MAC” on the X axis.

Comment: Line 541-542: Please consider changing “and enhances” to “and enhancement of”.

Response: This has been corrected, “and enhances” was revised to “and enhancement of”

Comment: Figure 7:
a. Are graphs B and C based on Western Blot (not shown) or immunofluorescent images (A). If based on Western Blot then please add blot images. If based on immunofluorescent then the almost doubling of the expression noted in “B” (MET vs CTRL) does not appear to be supported by the image in “A”.

Response: The graphs for Figure 7 are quantified from the captured immunofluorescent images. The data from the immunofluorescent images was re-quantified and reanalyzed to provide a revised graphical representation.

Comment: Lines 575 ot 577:
a. “Bactericidal activity” (used later in the text) could be considered instead of “bacterial killing”.

Response: This has been revised, we show that activation of PGC-1α enhances phagocytosis and bactericidal activity in NTM infected macrophages.

Comment: Line 579: Please consider “involved” instead of “functioning”.

Response: This is edited to, “the immune response generated against NTM infections encompasses multiple cell types, however macrophages are the primary cells involved in initial defense against inhaled mycobacteria, making their activity indispensable for an efficient immune response”

Comment: Line 612: Please consider removing “our”.

Response: “our” has been removed

Comment: Line 614: Please consider changing “resulting” to “results” and the “ΔΨm” to “MMP” as mentioned earlier in the manuscript.

Response: revised to “In accordance with other literature, MAB and MAC induce mitochondrial damage in macrophages that result in induced deficits in integrity of both mtDNA and MMP.”

Comment: Line 618: Please either consider removing “and immune cells” or adding “perhaps other” before it.

Response: Deficits in energy production and mitochondrial activity negatively impact the ability of macrophages and perhaps other immune cells to effectively combat bacterial infections.

Comment: Lines 631-632: Please consider minor revision for clarity.

Response: this has been revised, “ In addition to control over mitochondrial biogenesis, PGC-1α exerts multiple beneficial effects in immune cells, including expression of cytoprotective and antioxidant proteins.

Comment: Lines 650-651: Please consider revision/breaking the sentence.

Response: this has been revised, Our data show that NTM infections in vitro downregulate both PGC-1α and TFAM, two critical determinants of mitochondrial function that can impact the immune function of macrophages.

Comment: Lines 672-674: Please consider revision for better clarity.

Response: Sentences were revised to: “Metformin has been shown to reduce the severity of bacterial infections in preclinical and clinical retrospective studies, although the exact mechanisms remain elusive.The potential mechanisms by which Metformin exerts beneficial effects and rescues mitochondrial bioenergetics are related to its activation of AMPK and downstream activation of PGC-1α.”

Comment: Line 691: Please consider adding “and” after “bioenergetics,”.

Response: The wording was modified to read “In conclusion, our study shows that NTM infected macrophages exhibit mitochondrial damage with impaired bioenergetics and increased ROS production.”
