# Supplementary material for: PGC-1α activation to enhance macrophage immune function in mycobacterial infections
Source: PLoS One. 2025 Feb 6;20(2):e0310908. doi: 10.1371/journal.pone.0310908 (PMC11801632; doi:10.1371/journal.pone.0310908)
Supplement: Attachment — Submitted filename: Response to reviewers Jan 30 2024.docx [file pone.0310908.s004.docx]

**Reviewer #1:**

1) **Comment:** This manuscript, titled 'PGC-1α Activation to Enhance Macrophage Immune Function in Mycobacterial Infections,' aimed to investigate the impact of NTM infections on host macrophage mitochondria. Importantly, NTM disrupted the ability of macrophages to generate functional mitochondria by attenuating PGC-1α and TFAM, which were subsequently restored by PGC-1α pharmacologic activators. However, the in vivo studies lack solid evidence to conclusively prove that the altered expression of PGC-1α or TFAM, caused by NTM, occurred in macrophages.

**Response:** We appreciate the reviewer comment about the significance of our work, we agree that the in vivo model does not necessarily reflect the expression of the mitochondrial proteins only in the macrophages. They are probably a combination of multiple cell types. The purpose of the in vivo experiment is to demonstrate that the expression of the mitochondrial proteins is altered in the lung. We do agree that more in depth studies are needed to define the in vivo role of macrophages in the lungs. In future studies, we plan to study the in vivo model in depth, however, it is beyond the scope of the current manuscript.

2) **Comment:** The fluorescence images are obscure, as evidenced in Fig. 1C, Fig. 3K, Fig. 4K, and Fig. 4L.

**Response:** We have included labeling with the colors that are represented by the fluorescent images. This provides the explanation for the images; this is now included in the results and figure legends.

**Reviewer #2:**

**Comment:** This is a nice manuscript and worthy of publication. However, appreciate author’s comments/responses to the following points. We appreciate the reviewer comments and have addressed the concerns raised.

1. **Comment:** In general, language requires some revision and editing particularly in the “Discussion” section which requires significant revision for better clarity and removal of redundant and repetitive sentences.

**Response:** We appreciate the comment and have now carefully edited the manuscript for language and revised the discussion section for brevity and clarity (Lines 570-705)

2. **Comment:** Line 153: What dose of oral metformin in an average human would be equivalent to the final concentration of 2 mM metformin mentioned in the manuscript. Please add to the manuscript.

**Response:** This is an important point, thank you for raising this issue. Metformin dosages prescribed for diabetes range up to a maximum of 2.5 g (35 mg/kg) per day, which corresponds to blood sera levels of 10-20 µM. Pharmacokinetic evidence shows metformin’s action in the mitochondria occurs through a gradual build-up in the inner mitochondrial matrix, which can have concentrations orders of magnitude higher than serum levels. In one study, a serum concentration of 10 µM was correlated to mitochondrial concentrations 100 times higher. Thus, the elevated metformin concentrations used in this study and others correlates to intramolecular concentration of metformin in vivo.
3. **Comment:** Line 182: Is the correct number 15,000 RPM and not 150,000?

**Response:** Correct, the centrifugation speed is “15,000 RPM” (Line 182), this has been corrected

4. **Comment:** Lines 245, 293, 303: Please specify what the cells are treated with?

**Response:** We have now included specifics about drug treatments and concentrations. Cells were treated with metformin [2 mM], ZLN005 [2 µM], and SR-18292 [10 µM] (Line 245, 293, 305),

5. **Comment:** Figure 1C and 1D:
a. The MitoSOX staining for CTRL seems to be nothing (at least not visible on the provided image) where as mentioned as about 1C on Figure 1D.

**Response:** This figure has now been replaced.

b. MitoSox-based flow cytometry could be considered as an alternative

**Response:** We agree that flow cytometry can be alternative, however, the reason for using fluorescent cell staining was to illustrate and visualize the progressive increase in MitoSOX fluorescence in macrophages during the initial stages of bacterial infection.

6. **Comment:** Figure 2: Whole lung tissue was used for this experiment and thus it is not clear if the results seen are due to changes in macrophages, other cells, or both. This is important as the authors have previously shown that PGC-1a overexpression, resveratrol, and metformin enhance host bronchial epithelial cell mitochondrial function and improve epithelial innate response to P. aeruginosa. Showing the changes specifically in macrophages (by cell sorting or staining) will be important as they are the focus of the manuscript.

**Response:** We agree with the reviewer the in vivo experiments do not discriminate between the cell types are demonstrating the upregulation. We agree that the in vivo model does not necessarily reflect the expression of the mitochondrial proteins only in the macrophages. They are probably a combination of multiple cell types. The purpose of the in vivo experiment is to demonstrate that the expression of the mitochondrial proteins is altered in the whole lung. We do agree that more in depth studies are needed to define the in vivo role of macrophages in the lungs. In future studies, we plan to study the in vivo model in depth, however, it is beyond the scope of the current manuscript since the focus of the current manuscript is to investigate the effects on macrophages in vitro. While we cannot be certain the effect seen was due to cells other than alveolar macrophages, the in vivo experiments demonstrate changes in the whole lung tissue. We agree that in future experiments, it would be beneficial to isolate macrophages from the lung tissue to determine their specific influence on PGC-1α expression during NTM infection.

7. **Comment:** Figure 3 related:
a. There is an issue with discrepancy between the text (line 391 to 403) and figures 3A to 3H where the text states “MAB-mediated” but figures show “MAC”. b. However, Figures 3I and 3J, 3K mention MAB. Please reconcile.

7a-d. **Response:** We regret the oversight in labeling, figures and captions, are all revised.

e. Also, the morphology of the cells in the bottom row of figure 3K appear different from the rest. In our experience, the morphology of cells (roundish) in the 3 top rows is more in line with monocytes while the morphology in the bottom row is more in line with activated macrophages. Appreciate authors’ input.

**Response:** We appreciate the comment, the immunocytochemistry images are that of macrophages, which exhibit both circular and irregular/stellate shaped morphology.

**Comment:** Pre-treatment with ZLN005 was used in this experiment, would treatment after MAB or MAC infection produce similar results and be as effective? This is of clinical importance as treatment is usually initiated after the infection.

**Response:** We agree that treatment post infection would have translational implications, these experiments need careful dose response and time course experiments, are ongoing and will be tested in the future.

8. **Comment:** Figure 4 related: Would treatment with metformin after the infection produce the similar results?

**Response:** We anticipate similar results with metformin post treatment, since it is a potent PGC-1α activator.

9. **Comment:** Figure 5: . SR (I assume stands for SR-18292) is mentioned in the figure but no explanation in the text or figure legend.

**Response:** Correct, we have included this in the figure caption SR-18292 and in the abbreviation SR. (Line 520)

10. **Comment:** Line 492: Please clear the track changes (please remove “and macrophages” at the end of the sentence.

**Response:** Thank you, we have corrected this. “and macrophages” was deleted from sentence. (Line 492).

11. **Comment:** Lines 521-523: Please revise the sentence for better clarity

**Response:** We have revised lines 521-523 and made corrections for better clarity.

12. **Comment:** Lines 532-534: Given that TFAM is downstream from PGC-1a (as also mentioned in the manuscript) why SR-18292 had no measurable effect on TFAM despite significantly reducing PGC-1a expression?
**Response:** There are multiple signaling pathways and transcription factors involved with the activation and regulation of PGC-1α and TFAM, and both can be activated through PPARγ signaling. Thus there may be other potential pathways for activation of TFAM, additionally, post transcriptional modifications may be involved in differential gene expression between PGC-1α and TFAM.

13. **Comment:** Line 596: Please add “(mitochondrial membrane potential)” after ΔΨm.

**Response:** Thank you, this has been corrected.

14. **Comment:** A figure depicting the pathways and findings will be a welcomed addition

**Response:** We appreciate the suggestion and have added a figure (Figure 8).
