# Supplementary material for: PGC-1α activation to enhance macrophage immune function in mycobacterial infections
Source: PLoS One. 2025 Feb 6;20(2):e0310908. doi: 10.1371/journal.pone.0310908 (PMC11801632; doi:10.1371/journal.pone.0310908)
Supplement: Attachment — Submitted filename: Response to reviewers PlosOne June 20 2024.docx [file pone.0310908.s006.docx]

We appreciate the reviewers detailed review and comments for correction. We have incorporated all the changes.

**Reviewer Two**

**Comment**

1. Ethics section, lines 320-322 of the first revised version has been omitted from the second (current) revised version. Please clarify.

Response

The studies do not have any human specimens therefore the ethics section is omitted

**Comment**

2. Line 355 of the current version (ppargc1a) should be in italic

**Response**

This has been reformatted

**Comment**

3. Line 357 of the current version (tfam) should be in italic

**Response:**

This has been formatted

**Comment:**

4. Figure 2 (2A to 2L) corresponding to data related to ZLN005 is missing.

**Response:**

ZLN005 has been added
