# Supplementary material for: PGC-1α activation to enhance macrophage immune function in mycobacterial infections
Source: PLoS One. 2025 Feb 6;20(2):e0310908. doi: 10.1371/journal.pone.0310908 (PMC11801632; doi:10.1371/journal.pone.0310908)
Supplement: Attachment — Submitted filename: Response to Reviewers.docx [file pone.0310908.s007.docx]

All requested changes have been incorporated into the manuscript and figure files.

**Reviewer Two**

Comment:

Reviewer #2: Although this manuscript is interesting and worthy of publication, it appears that the manuscript is not carefully prepared evident by multiple errors over several revisions which has unfortunately continued even in the most recent version.

Figure 2, is not incorporated into the figures and is separately added out of place at the end of the figures. The quality of the figure 2 is not great and apparently does not have a corresponding TIFF file (or at least this reviewer could not download it). Therefore, as is, figure 2 is not suitable for publication. Please make sure that a good quality figure is added and properly incorporated in its right place among figures

Response:

Figure 2 was reformatted to a higher quality TIFF file and uploaded in the proper sequence.

Comment:

Line 363: It seems that “and TFAM (FIG 2F)” should be “TFAM (FIG 2G)”

Response:

The proper figure title was added

Comment:

Line 367: It seems that “PGC-1a (FIG 2G)” should be “PGC-1a (FIG 2F)”

Response:

The proper figure title was added
